# The Orbital Destruction Intensity Classification—An Easy-to-Use, Numerical Scale for Assessing the Severity of Orbital Fractures

**DOI:** 10.3390/jcm14113826

**Published:** 2025-05-29

**Authors:** Kacper Galant, Marcin Kozakiewicz, Agata Ciosek, Katarzyna Bogusiak, Izabela Gabryelczak

**Affiliations:** 1 Student Scientific Club of Maxillofacial Surgery, Medical University of Lodz, 113 Żeromskiego Str., 90-549 Lodz, Poland; agata.ciosek@stud.umed.lodz.pl; 2 Department of Maxillofacial Surgery, Medical University of Lodz, ul. Żeromskiego 113, 90-459 Lodz, Poland; marcin.kozakiewicz@umed.pl (M.K.); katarzyna.bogusiak@umed.pl (K.B.); gabryelczakizabela@gmail.com (I.G.)

**Keywords:** orbit, orbital fractures, injury, classification, diagnostic imaging, fracture classification

## Abstract

**Background/Objectives**: Orbital fractures are a very serious problem due to the close location of the eyeball and a direct path to brain injuries, which is associated with serious consequences. This study aims to assess the usefulness of the Orbital Destruction Intensity (ODI) scale. Additionally, this article includes elements of an epidemiological study. **Methods**: A retrospective study of 160 patients admitted to the Department of Maxillofacial Surgery in Łódź (Poland) between January 2021 and June 2024 was conducted. In this study, general patient information (gender, age), details about the injuries (cause, affected orbit, accompanying symptoms), diagnosis (ODI scale, pathological classification), and treatment were assessed. Analysis of the distribution of features and regression analysis was performed in the case of quantitative data. To compare the assessment of the impact of a categorical variable on a quantitative variable, the Kruskal–Wallis test was used. A p-value of less than 0.05 was considered statistically significant. **Results**: The main cause of the accident was assault, which accounted for 39% of cases. An X-ray examination showed that patients had an average ODI score of 2.92 ± 1.69. Patients with low ODI scores mostly had isolated fractures of the orbital floor. As ODI scores increased, zygomaticomaxillary complex (ZMCO) fractures became more common as an additional fracture (*p* < 0.05). For patients with low ODI scores, treatment generally involves reconstructing the orbital wall with titanium mesh. For those with higher ODI scores, treatment may include microplate osteosynthesis or a combination of both methods (*p* < 0.05). **Conclusions**: A correlation was observed between the diagnosis based on ODI, anatomical classification, and the treatment provided. This relationship is related to the nature of the ODI scale, as, when the severity of the injury increases, additional anatomical structures (walls or rims of the orbit) are included.

## 1. Introduction

Among all maxillofacial injuries, orbital trauma has become increasingly common, accounting for approximately 25–60.1% [1,2]. The most common causes of injury are road traffic accidents (31.2–37.8%) and assaults (17.23–39.3%) [3,4,5].

**Figure 1:**
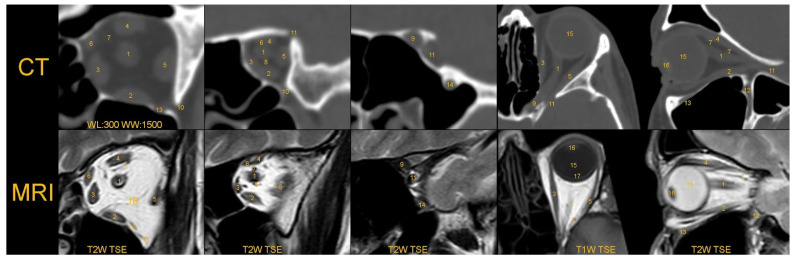
Important anatomical structures observed in the eye socket in two principal imaging methods. 1: nervus opticus, musculi recti orbitae: 2: inferior, 3-medialis, 4: superior, 5: lateralis, 6: musculus obliquus superior, 7: vena ophthalmica superior, 8: arteria ophthalmica, 9: canalis opticus, 10: fissura orbitalis inferior, 11: fissura orbitalis superior, 12: fossa pterygopatina, 13: canalis infraorbitalis, 14: foramen rotundum, 15: corpus vitreum oculi, 16: lenticula oculi, 17: retina, 18: systema ligamentaris corporis adiposi orbitae. Note the complexity of the fine structure of the ligamentous system in the fat body of the orbit revealed by MRI and the ability to observe the course of the retina in a T1-weighted sequence. Abbreviations: CT: computed tomography, MRI: magnetic resonance imaging, WL: window level in Housfield units, WW: window width in Housfield units, T2W_TSE: image of T2-weighted turbo spin echo, T1W: weighted image.

The orbit contains key structures such as the eyeball, oculomotor muscles, optic nerve, ophthalmic artery, the branches of the V1 division of the trigeminal nerve, and trochlear nerve (Figure 1). It comprises seven bones: frontal, sphenoid, maxilla, palatine, zygomatic, ethmoid, and lacrimal.

The canal in the lower orbital wall contains the infraorbital nerve, which may be damaged in more than half of cases, leading to sensory impairment [3,4]. The most important aspect of treatment is the early diagnosis and preservation of the globe, since orbital trauma is known to be the second most common cause of blindness [6], which, along with facial aesthetic disorders [7,8,9], significantly affects the patient’s overall well-being. Orbital injuries often present with symptoms such as swelling (87.88%), damage to the infraorbital nerve (26.3–53.41%), double vision (44.5–72.35%), enophthalmos (36.9–41.29%), and limited eye movement (25.2–51.70%) [3,4].

These symptoms often require orbital reconstruction. Orbital fractures are the most common cause of surgical interventions and account for 44% of all fracture-related procedures [10]. In surgical treatment, titanium meshes (55.11%) [3] or bioresorbable implants (38.11%) [4] are often used. The primary goal is to restore the bony integrity of the orbit and obtain the appropriate orbital volume.

The Orbital Destruction Intensity (ODI) scale was created and introduced by Kozakiewicz et al. in 2011 [11]. This scale helps assess therapeutic and implantological needs in the case of orbital fractures. The ODI classification is a simple and clear tool that can especially help young clinicians to assess the severity of fractures and decide on the best treatment approach. Precise assessment of the severity of the injury is essential for choosing the right treatment method and predicting the patient’s prognosis.

This study aims to validate the ODI score based on its correlations with anatomical classification and clinical outcomes of orbital fractures. We hypothesize that higher ODI scores are associated with more complex orbital injuries that require more extensive surgical interventions.

## 2. Materials and Methods

### 2.1. Data Collection Process

We conducted a retrospective single-center observational study involving 160 patients admitted to the Maxillofacial Surgery Department at the Medical University of Łódź, Poland, between January 2022 and June 2024. Patients were identified in the database using the following “ICD-10-CM’s 2024” codes: S02.3 (Fracture of orbital floor) and S02.4 (Fracture of the malar and maxillary bones), which capture injuries related to the orbit and adjacent structures, and verified in dicom file viewer RadiAnt (https://www.radiantviewer.com/en/ access date 14 December 2024).

From the database, various parameters were extracted, including (1) age, (2) gender, (3) date, (4) cause of the incident, (5) time of admission, (6) treatment method, (7) duration of hospitalization, (8) visual system symptoms, (9) trigeminal nerve disorders, (10) dental injuries, (11) intoxicant-related injuries, (12) loss of consciousness, (13) orbital hematomas, and (14) maxillary sinus lesions.

The study included the results of strabological examinations. In the case of significantly severe and/or persistent symptoms, such as diplopia, cooperation was established with specialists in this field—ophthalmologists. Strabological data were obtained from both the medical history (assessed by a specialist in maxillofacial surgery) and/or the previously mentioned examination (performed by an ophthalmologist). If such information was not documented in the patient’s card, it was assumed that it was not reported by the patient and/or the attending physician did not observe any deviations. Since the results of the strabological assessments are not the subject of this publication, only the most significant and common complications are included.

The data were collected by two authors (K.G. and A.C.), and tables were prepared using Google Sheets software (Google LLC, Mountain View, CA, USA; accessed September 2024) and Microsoft Excel (version 2301, Microsoft Office Professional Plus 2016; Microsoft Corporation, Redmond, WA, USA) Data regarding post-operative diplopia were obtained by checking the follow-up history. If the patient did not attend the follow-up visit, their telephone number was taken from the chart, and an attempt was made to contact and receive information.

Computed tomography (CT) scans were performed in all patients upon admission to the hospital. The retrospective evaluation using the ODI scale was based on these imaging studies or, when applicable, their full radiological descriptions, regardless of whether the patient underwent surgical or conservative treatment.

In cases of doubt regarding fracture classification or patient eligibility, one of the authors (M.K.) made the final decision based on a thorough review of CT scans and clinical records.

### 2.2. Included Classification

Initially, the Wanyura [12] and Le Fort classifications, commonly used in the Polish literature, were used to develop the anatomical classification. However, due to terminological discrepancies with the English-language literature, the diagnoses were converted according to the following rules (Table 1). No conversion of ZJO or DON fractures (Wanyura’s classification, which corresponds to naso-orbito-ethmoid (NOE) fractures) was performed, as these types were not diagnosed in the study group.

**Figure 2:**
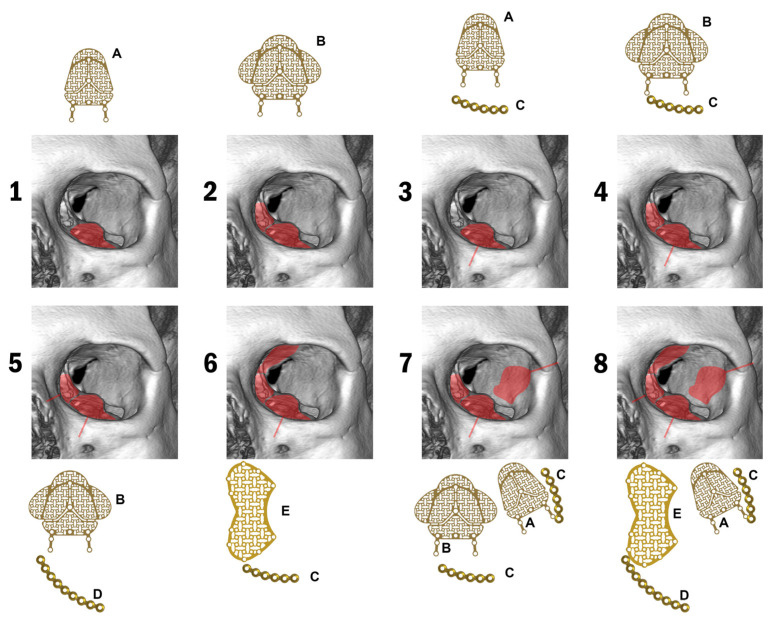
Illustration showing the distribution of injury according to the ODI scale. Rising numbers in the ODI also determine increasing implant needs in the orbit. For treatment in ODI 1, a standard mesh is sufficient (A: ref. 3.6885.100 www.chm.eu access date 19 May 2025), for restoration of two damaged walls, expanded meshes are provided (B: ref. 3.7359.100 one wing can be cut from it and the lower and medial walls can be restored). When there are rim fractures, 1.5 mm system microplates are used first (C: ref. 3.3474.006 and D: 3.3474.009). A dedicated mesh (E: ref. 3.6874.001) can be used to reconstruct three walls, or the upper wall can be placed without reconstruction. Red area/line—area affected by the fracture/fracture of the orbital rim. The number corresponds to the number in the ODI scale.

Patients were categorized according to the ODI scale based on a retrospective analysis of archived CT scans and anatomical diagnoses made during hospital shifts [11] (Table 2, Figure 2). This scale allows for the stratification of patients by injury severity and implant needs. It describes the number of affected orbital walls and rims in different configurations.

### 2.3. Eligibility Criteria

Our study included only hospitalized patients admitted to the Maxillofacial Surgery Department; patients treated on an outpatient basis were excluded from the study. Included were patients with a confirmed diagnosis of orbital fracture (ICD-10 S02.3 or S02.4) and complete medical history with available imaging data.

Exclusion criteria for this study were: the absence of orbital trauma and incomplete medical records (missing X-ray or CT scan of the facial skeleton or lack of a radiologist’s report) (Table 3).

### 2.4. Statistical Analysis

Statistical analysis was performed by one of the authors (M.K.) using Statgraphics Centurion XVI (StarPoint Technologies. Inc., The Plains, VA, USA). Statistical analysis includes feature distribution evaluation, and analysis of regression was applied to assess the relationship between quantitative variables. The Kruskal–Wallis test was applied for evaluation of the categorical variables’ (the factor) influence on quantitative variables. Next, a multiple comparison procedure was used to determine which means were significantly different. The method discriminates among the variables in the Fisher’s least significant difference (LSD) procedure. The relationship between qualitative features was assessed using the χ^2^ independence test. The difference was considered significant if *p* < 0.05.

## 3. Results

### 3.1. General Information

Over the past 2.5 years, 160 cases have been reported, of which 25% (n = 40) were women and 75% (n = 120) were men, with an average age of 40 years ±18.

The time after which patients reported to the hospital ward was measured, with an average of less than 24 h and a maximum of 19 days. Patients were treated for slightly over 3 days ± 1 day.

More than one in four patients (28.9%; n = 46) experienced loss of consciousness.

Data on age, diagnosis based on the ODI classification, time to register, and the duration of hospitalization, as well as their statistics, are presented in Table 4.

### 3.2. Main Causes of Fractures

**Figure 3:**
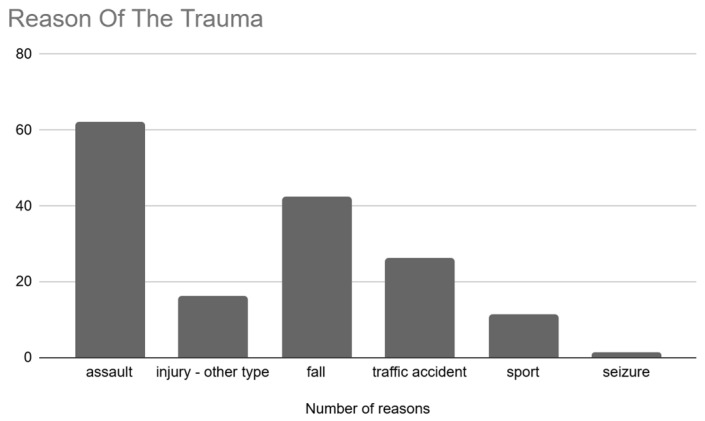
Main causes of the fractures.

The main causes of orbital fractures were assault, 39.2% (n = 62), falls, 26.6% (n = 42), and road accidents, 16.5% (n = 26) (Figure 3).

Low-value injuries in the ODI scale were diagnosed in patients who suffered from low-energy injuries like assaults; together with the growth of diagnosis values, the energy of the injuries increased (traffic accidents) (*p* < 0.05).

### 3.3. Types of Fractures/Diagnosis

The distribution of affected orbits is almost even, but with a predominance of left orbit injuries (left vs. right orbit vs. both; 50% vs. 46.9% vs. 3.1%).

**Figure 4:**
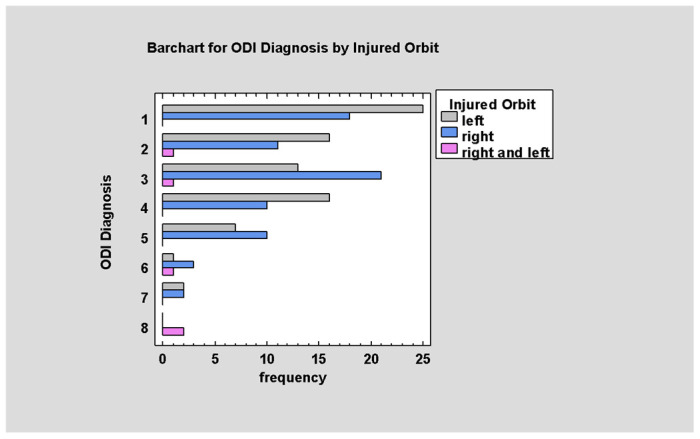
ODI Diagnosis by Injured Orbit.

Left orbit injuries are predominant in lower numbers of the ODI scale. With increasing value in the ODI scale, this domination declines (*p* < 0.05; Figure 4).

**Figure 5:**
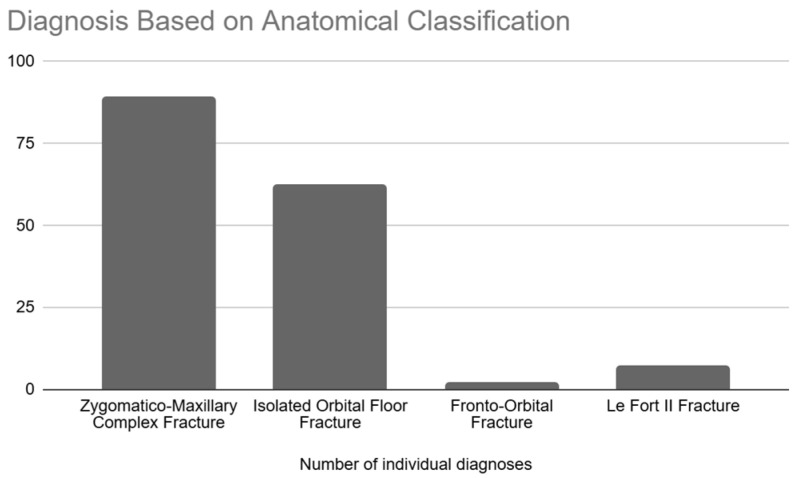
Diagnosis based on anatomical classification.

The number of individual injuries verified according to anatomical classification is presented in Figure 5. ZMCO fractures were the most prevalent, constituting over half of the cases at 55.6% (n = 89), while the least common were fronto-orbital fractures, accounting for 1.3% of all cases (n = 2).

While making a diagnosis based on the ODI classification, an average result of 2.92 ± 1.69 was obtained (Table 4).

**Figure 6:**
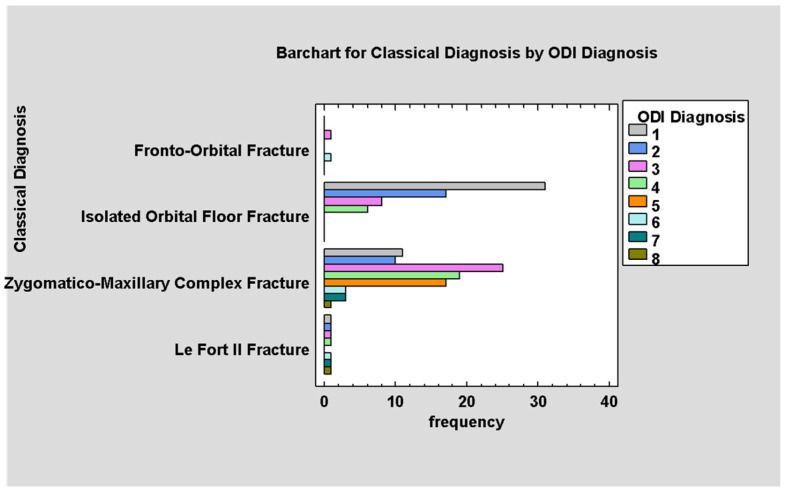
Classical Diagnosis by ODI.

There is a strong relation between the diagnosis based on the anatomical scale and ODI. Higher ODI values correspond to ZMCO fractures and lower values to isolated orbital floor fractures (*p* < 0.05; Figure 6).

### 3.4. Intoxicants

Some patients who suffered orbital fractures were under the influence of intoxicants, such as alcohol 34% (n = 54) or both alcohol and drugs 1.3% (n = 2).

**Figure 7:**
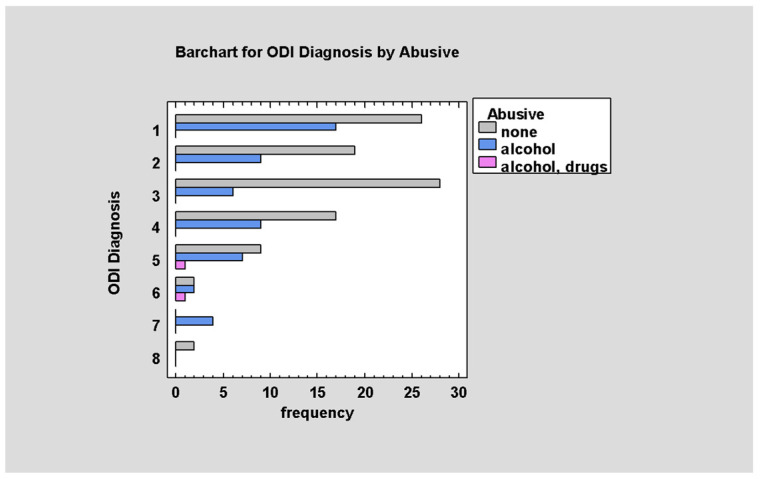
ODI by intoxicant.

Patients under the influence of intoxicants reach higher scores according to the ODI scale compared with sober patients in which those values are lower (*p* < 0.05; Figure 7).

### 3.5. Post-Traumatic Complications

#### 3.5.1. Periorbital Hematoma

Patients were more likely to develop a right periorbital hematoma compared to the left one (n = 57 vs. n = 53; 35.36% vs. 33.13%). Approximately 25% (n = 40) of cases had no hematoma at all, and in 6.25% (n = 10), a binocular hematoma occurred.

The incident of periorbital hematoma is not related to the diagnosis made based on the ODI scale (*p* > 0.05).

#### 3.5.2. Maxillary Sinus Lesions

In the majority of cases (95%; n = 152), a connection with the maxillary sinus was formed, leading to the entrapment of oculomotor muscles, the development of hematoma, or other post-traumatic changes.

#### 3.5.3. Eye Globe Position

One hundred and thirty-eight (86.25%) patients did not experience eyeball position abnormalities. Exophthalmia developed in only 16 (10%) patients and enophthalmia in 6 (3.75%) patients.

#### 3.5.4. Trigeminal Nerve Disturbances

In 61.9% of cases, damage to the trigeminal nerve branches occurred. Of these, 96.97% involved the maxillary branch (V2) of the trigeminal nerve, specifically the infraorbital branch.

**Figure 8:**
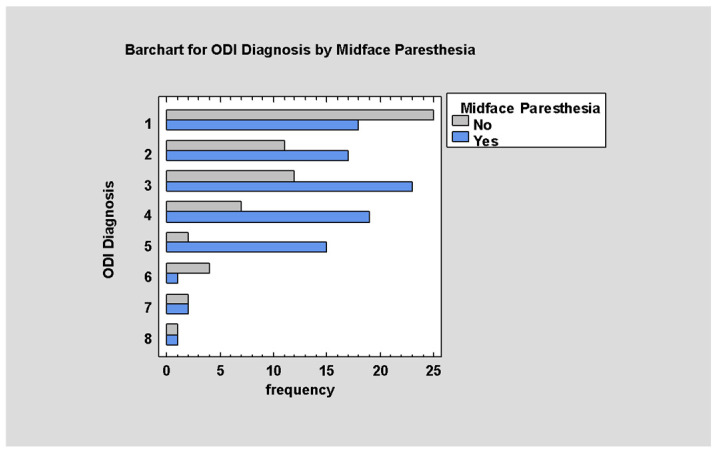
ODI by Midface Paresthesia.

Post-traumatic sensory disturbances are accompanied by medium-value ODI (*p* < 0.05; Figure 8).

#### 3.5.5. Teeth Injury

Among all patients, 17.5% (n = 28) developed malocclusion, however, in most cases (81.88%; n = 131) this disorder did not occur. Only eight patients experienced dental trauma.

Higher ODI values, which stand for increased trauma energy, are accompanied by dental injuries (*p* < 0.05).

#### 3.5.6. Post-Traumatic Diplopia

Diplopia was considered in two aspects: pre-op. and post-op. In 33.1% (n = 53) of patients, diplopia developed as a result of trauma, and in 28.1% (n = 45), it also occurred after surgery.

##### Post-Traumatic Diplopia According to the ODI Scale

**Figure 9:**
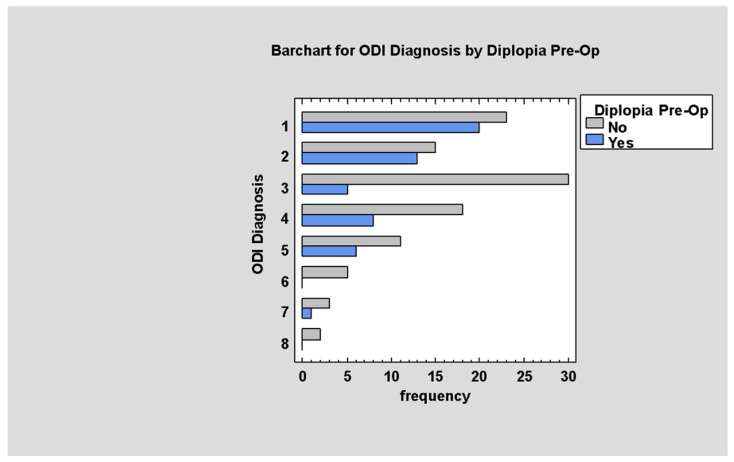
ODI by Diplopia Pre-op.

Most patients with low–medium ODI had diplopia pre-op. (*p* < 0.05; Figure 9).

**Figure 10:**
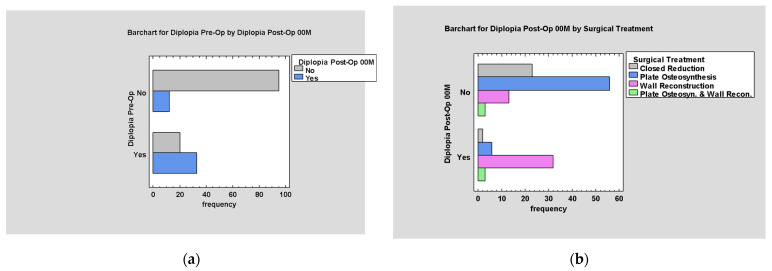
The graphs show the relationship between Post-Op. diplopia and: (**a**) Pre-Op. diplopia; (**b**) the type of treatment used.

Post-operative diplopia was mainly persistent diplopia from before surgery (*p* < 0.05; Figure 10a). It mainly accompanies wall reconstruction procedures. The absence of diplopia occurred in the vast majority of cases when plate osteosynthesis or closed reduction procedures were performed (*p* < 0.05; Figure 10b).

**Figure 11:**
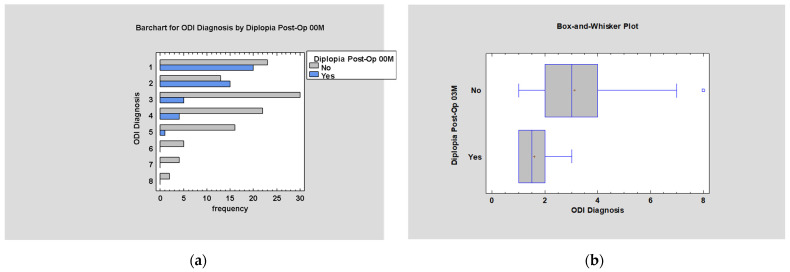
(**a**) ODI by Diplopia Post-Op. 00M; (**b**) ODI by Diplopia Post-Op. 03M.

Diplopia appears mainly in cases of low values of the ODI scale in early post-operative examination (*p* < 0.05; Figure 11a).

The most significant decrease in diplopia incidents within 3 months was noted in patients with medium values of the ODI (*p* < 0.005; Figure 11b).

**Figure 12:**
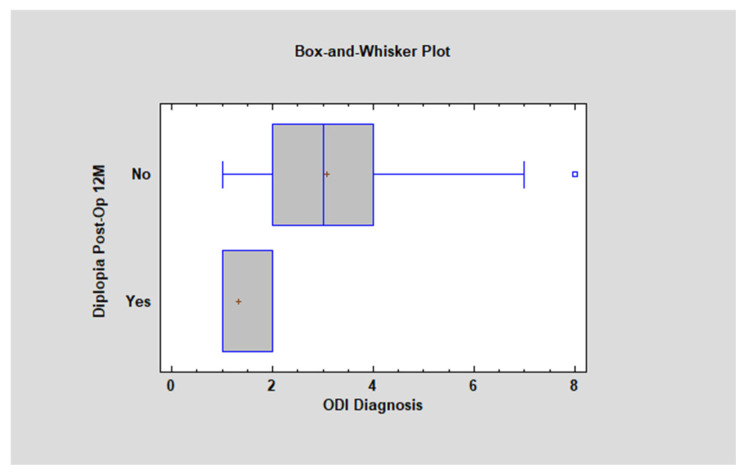
Diplopia Post–Op. 12M by ODI diagnosis. Plus sign—mean value; square—extreme value; vertical line in the box—median.

Despite treatment for low ODI values, diplopia persists for over 12 months (*p* = 0.06, Figure 12).

##### Post-Traumatic Diplopia According to Anatomical Classification

Pre-op. diplopia is particularly common in orbital floor fractures compared to others, however, in most cases, ZMCO fractures can also lead to double vision with a frequency of 1:4 patients (*p* < 0.05).

**Figure 13:**
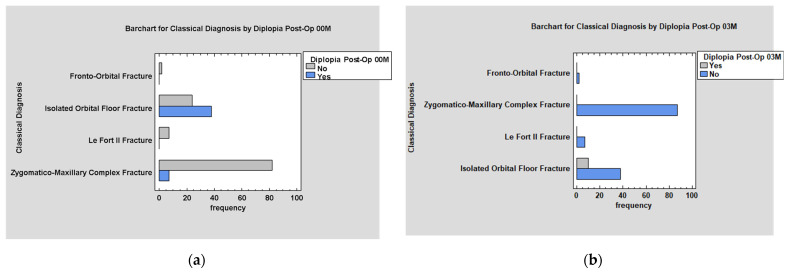
(**a**) Classical Diagnosis by Diplopia Post-Op. 00M; (**b**) Classical Diagnosis by Diplopia Post-Op. 03M.

Treatment of diplopia associated with ZMCO fractures is more effective than treatment of isolated orbital floor fracture at both the 3- and 12-month follow-up examinations (*p* < 0.05; Figure 13a,b).

### 3.6. Treatment

Of patients, 86.25% underwent surgical treatment, and the rest received conservative treatment (Table 5). According to the surgical procedures, plate osteosynthesis was the most prevalent and was performed in 44.9% of cases (Table 6).

**Figure 14:**
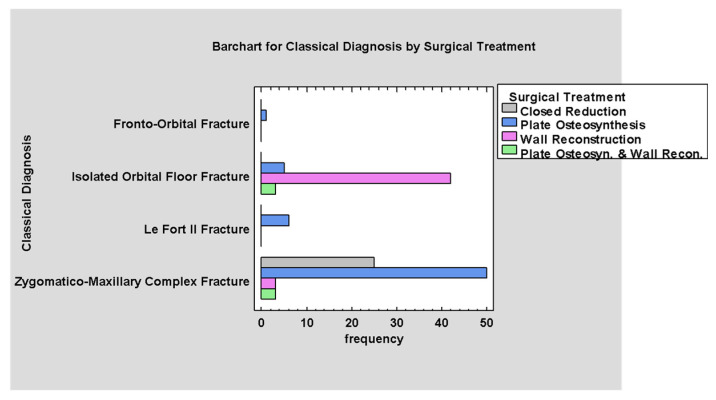
Classical Diagnosis by Surgical Treatment.

There is a relation between the anatomical diagnosis and the method of treatment. Isolated orbital fractures are treated mainly with wall reconstruction and ZMCO fracture with closed reduction or microplate osteosynthesis (*p* < 0.05; Figure 14).

**Figure 15:**
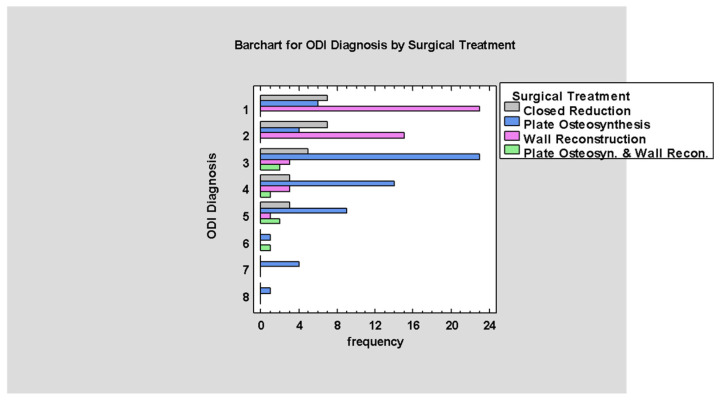
ODI by Surgical Treatment.

Analysis of Figure 15 shows a relation between higher values of the ODI scale and treatment by osteosynthesis, as when the values of the ODI scale increase patients treated with wall reconstruction decrease and the need for microplate osteosynthesis or both methods simultaneously increases (*p* < 0.05; Figure 15).

## 4. Discussion

This research proposed and evaluated a novel quantitative classification system—Orbital Destruction Intensity (ODI)—designed to provide a simple and objective assessment of orbital fractures. Findings from this study highlight a link between the ODI score and relevant fracture characteristics, such as the extent of orbital trauma, or association with significant post-traumatic complications. Unlike current descriptive systems, the ODI classification stands out as a vital complement to them. The results support the validity of the ODI scale as a potentially useful tool in both diagnostic and therapeutic planning contexts, which could be applied worldwide with further validation.

### 4.1. The Extent of Orbital Trauma and Its Association with the ODI Classification

In this study, the values on the ODI scale reflected the size and extent of one’s injury, since its score is primarily based on the number and location of fractures in the orbital walls. This study demonstrated a link between the ODI score, the number of fractured orbital walls, and the type of fracture related to it according to the anatomical classification. Patients who experienced high-energy trauma, like traffic accidents, which often involve inelastic impacts that cause significant deformation [13], suffered from more complex fractures, like zygomaticomaxillary complex fracture (ZMCF), and had higher values on the scale (e.g., 4–6), requiring prompt and complex management. In contrast, patients with low-energy trauma, such as falls, had lower ODI scores (e.g., 1–2) and were mostly diagnosed with isolated orbital fractures (IOFs). Therefore, this validates the ODI classification as a potentially reliable indicator of fracture distribution and severity, thereby supporting a more accurate and quicker selection of the most appropriate treatment method.

### 4.2. Post-Traumatic Complications and Their Occurrence According to ODI Classification

Another key finding from this study was the notable correlation between ODI scores and clinical complications commonly associated with orbital trauma.

#### 4.2.1. Post-Traumatic Diplopia

One of the most severe and common complications of orbital fractures is the presence of post-traumatic diplopia [14,15,16]. In this study, double vision occurred in most cases as a result of trauma (33.1%; n = 53), rather than after surgical treatment (28.1%; n = 45). According to research findings, patients with IOF experienced diplopia more often, while patients with ZMCF showed more trauma-related symptoms, such as subconjunctival and retrobulbar hemorrhages, along with relative afferent pupil defects [17]. Considering this association, using the ODI scale values, patients with lower values (e.g., 1–2), which were linked to IOF, experienced pre-operative diplopia frequently. This might be due to significant edema and hemorrhages around the eyes, which are typically associated with more complex fractures [17] that contribute to higher ODI values, such as ZMCF, preventing patients from fully recognizing the double vision [3]. Therefore, it is important to remember that edema and hematoma can either mask or emphasize the presence of post-traumatic diplopia [3].

#### 4.2.2. Connection with the Maxillary Sinus

Another serious complication of traumatic orbital fractures is the formation of a maxillary sinus fistula [18], which was also the most prevalent one in this study (95%; n = 152). This problem is particularly associated with lower scores and linked to IOF. In these types of fractures, the inferior wall is usually damaged, and a herniation of the orbital fat or inferior rectus muscle into the maxillary sinus occurs [3]. This could be due to the orbital floor being the weakest of the four walls and is easily fractured [19], making it one of the most common fracture sites in orbital trauma [20], and requires prompt treatment.

#### 4.2.3. Trigeminal Nerve Disturbances

Since orbital fractures most often involve the orbital floor, due to its vulnerability [19], the infraorbital nerve (ION), which adheres to it, may be easily damaged by trauma or during subsequent surgical interventions [21]. In this study, the second most common complication was trigeminal nerve disturbances (61.9%; n = 99), especially ones including the maxillary nerve (V2) and its infraorbital branches, which is also one of the frequent complications of facial trauma [3]. Post-traumatic sensory disturbances seem to be accompanied by middle values of the ODI scale (e.g., 3–5).

#### 4.2.4. Dental Trauma

Malocclusion was not the main post-traumatic complication (17.5%; n = 28), just as eyeball positioning abnormalities such as exophthalmia (10%; n = 16) or enophthalmia (3.75%; n = 6). However, higher values of the ODI scale were attributed to dental trauma. Trauma resulting in higher ODI is linked to higher energy and an extended area of injury, which can lead to damage in the lower parts of the maxilla, including alveolar processes.

#### 4.2.5. Periorbital Hematoma

On the other hand, there was no significant relationship between the occurrence of periorbital hematoma and values on the ODI scale, as either higher or lower scores do not indicate the occurrence of this complication, just as the presence of periorbital hematoma does not indicate a higher injury value. Therefore, this factor cannot be used as a diagnostic tool for orbital fractures.

#### 4.2.6. ODI Scale Values Concerning Post-Traumatic Complications

The results presented in this research underline the usefulness of the ODI scale as a probable tool for the initial post-traumatic patient evaluation. Patients with lower scores are more likely to develop pre-operative diplopia or herniation of the maxillary sinus, which should be treated immediately upon diagnosis or suspicion. In contrast, patients with higher scores, who may have typically experienced more severe, complex injury, have an increased likelihood of complications, such as trigeminal nerve dysfunction, dental trauma, and swelling, which can mask problems including pre-operative diplopia. Those patients will require multistage treatment and mitigation of health issues that mask other important post-traumatic complications.

### 4.3. Surgical Treatment According to ODI Classification

IOF is primarily treated with wall reconstruction using implants placed over the fracture site [22], whereas ZMCF is usually treated with closed reduction or microplate osteosynthesis [23,24,25]. In this study, the therapeutic interventions associated with the ODI scores corresponded to the procedures implemented based on the anatomical scales. Thus, low ODI values linked to IOFs were indeed treated with orbital reconstruction, and higher ODI values associated with ZMCF were treated with closed reduction or osteosynthesis. In cases with the highest ODI values (i.e., 6–8), patients are often in a severe general condition, requiring a personalized treatment approach. Those procedures should be divided into two stages: the first stage involves the use of microplate anastomoses, and the second stage involves orbital wall reconstruction once the patient’s condition has been stabilized.

Orbital fractures are challenging injuries as they may lead to serious, long-term complications, such as impairment or complete vision loss [26,27]. For this reason, prompt and appropriate intervention is crucial. The ODI classification provides a simple, easy-to-use numerical scale that allows for a rapid assessment of the severity and extent of the patient’s orbital injury. The obtained score allows the selection of the most appropriate treatment method, for example, lower ODI values are associated with less severe fractures that can be treated with wall reconstruction, intermediate values require both wall reconstruction and rim osteosynthesis, and higher values usually indicate more severe overall conditions, which require initial rim osteosynthesis to splint and stabilize facial wounds before reconstruction.

Figure 1 presents the treatment strategies for orbital fractures proposed by the authors, based on diagnoses made using the ODI scale, highlighting the importance of proper pre-operative qualification. The scale evaluated in this study may serve as a useful tool to support this decision-making process.

Another reason the ODI scale is crucial for treatment decision making is that on-call physicians often overlook additional orbital rim fractures (ODI 3 and 4 in IOF), focusing primarily on the damaged orbital floor. This may complicate orbital wall reconstruction due to the instability of the orbital rim. Thus, the ODI scale may play a key role in guiding surgical decisions, helping to avoid inappropriate treatment choices.

Additionally, ZMCF is an umbrella term covering various reconstructive needs for the orbit. The ODI classification is especially important in these cases, as most patients require specific variations of orbital wall reconstruction rather than just closed reduction of the displaced zygomatic bone.

### 4.4. Post-Operative Diplopia

According to the results, in an early post-operative examination, diplopia appeared mostly in patients with lower ODI, primarily those with IOF (21.25%; n = 34). These fractures require wall reconstruction and intervention within the eye socket, which tends to result in more cases of post-operative double vision compared to procedures for ZMCF. This type of fracture is typically managed with surgeries which do not involve manipulation within the orbit. Closed reduction in some cases of ZMCF is sufficient, and there is no need to access the orbital tissues. Improving the position of the zygomatic arch and the processes that constitute it results in subsequent tightening of the intraorbital periosteum and improvement of anatomical conditions without the need for a treatment using various types of meshes. This restores the original orbital contents, leading to better outcomes for diplopia reduction. Despite the treatment for low ODI values, diplopia persists for more than 12 months. IOF, in general, may lead to short-term diplopia as well as its long-term persistence, which, as previously mentioned, may be due to complications such as lower rectus muscle herniation and surgical interference in the eye socket, both of which have a relevant impact on the occurrence of diplopia [3].

Loba et al. revealed that, in most cases, double vision that persists after reconstructive surgery gradually resolves on its own in months after treatment. However, there are situations in which surgical intervention, such as strabismus surgery, may be essential to restore proper vision. The presence of double vision should be the main factor indicating further treatment. More than 60% of patients seem to have a complete or partial resolution or major reduction of double vision, which is not bothering them, especially while looking upward/in extreme gaze. Reoperation due to diplopia is necessary only in certain cases [28].

### 4.5. ODI Classification in Comparison to Other Widely Used Scales for Evaluating Midface Fractures

#### 4.5.1. Description of Commonly Used Scales

Various classification systems for the assessment of facial trauma are used in everyday clinical practice [29]. The most applied scales describing orbital fractures are the Arbeitsgemeinschaft für Osteosynthesefragen (AO) classification of craniomaxillofacial fracture, Zingg classification, and Jaquiéry classification. Another crucial one, but mainly referring to maxillary trauma, is the Le Fort classification. Those scales assess facial trauma based on location, complexity, and clinical relevance. The AO CMF classification offers a detailed, three-level numerical system categorizing fractures by region, subregion, and complexity [30,31]. The Zingg classification focuses on zygomaticomaxillary complex (ZMC) fractures, dividing them into three types (A–C) based on severity and displacement [32,33,34]. The Jaquiéry classification addresses orbital floor fractures, categorizing them into four types (I–IV) to help predict complications and guide reconstruction [29,35]. The Le Fort classification divides mostly midface fractures into three types and is essential for surgical planning or assessing functional impact, particularly involving the maxilla and nasal structures [36,37].

#### 4.5.2. Current Limitations of Commonly Used Classification Systems

Despite many advantages, the previously mentioned classifications present some limitations regarding orbital trauma. Although these systems are valuable for structural assessment and general categorization of maxillofacial injuries, they are largely descriptive and can be difficult for non-specialists, who lack the proper expertise to interpret. Importantly, apart from the Jaquiéry classification, most of these scales do not indicate the possibility of post-traumatic complications such as visual or sensory impairment, diplopia, loss of orbital volume, or soft tissue involvement—factors that are crucial for accurate prognosis and surgical decision making. The lack of quantitative parameters in most of these classifications also limits their ability to assess the severity of orbital displacement and predict complications such as enophthalmia or diplopia that significantly impact treatment selection. Therefore, despite their usefulness in categorizing fractures, these scales may not fully support the comprehensive evaluation and treatment of orbital fractures, especially in complex cases requiring tailored reconstructive strategies.

#### 4.5.3. ODI Classification and Its Application to Other Systems

When compared to other classification systems, the ODI scale stands out as an important complement to them. The ODI could provide a simple, numerical, and structured measure of orbital trauma, allowing for a better and quicker assessment of fracture severity and its impact on probable key functions such as diplopia, involvement of soft tissue or cranial nerve, and dental injury. It also stands out as an easy-to-use tool for different specialists. This aspect will be discussed in more detail in the following parts of the discussion.

### 4.6. Limitations

#### 4.6.1. Study Limitations

While this study provides a valuable insight into the application of a novel ODI classification system, certain limitations should be considered when interpreting the findings. This is single-center retrospective research conducted on a relatively small and undifferentiated group of participants. This may result in selection bias and limit the generalizability of the results. The findings may not fully represent broader populations or reflect clinical practices across different healthcare systems. Furthermore, the sample size was relatively small and consisted predominantly of patients with IOF or ZMCF. Additionally, during the study, not all possible types of orbital fractures, including naso-orbito-ethmoid (NOE) fracture, were noted. As a result, the performance of the ODI scale in more complex or rare fracture patterns remains uncertain. While post-operative complications such as diplopia were monitored, the study lacked long-term follow-up across all patient groups. Therefore, conclusions about the predictive value of the ODI scale for chronic outcomes should be interpreted with caution.

#### 4.6.2. The ODI Scale Limitations

Despite its promising potential, the ODI classification system also has limitations, which should be considered while obtaining its validity. Although the values on the ODI scale showed correlation with trauma severity and post-traumatic complication rates in this study, its reliability and accuracy have not yet been validated in external or multicenter settings. There is a need for further validation to prove its clinical utility. Furthermore, the ODI classification was established for the detailed evaluation of orbital trauma to ensure rapid management and prevent permanent complications. Therefore, in cases where orbital trauma is accompanied by multiple facial fractures (e.g., panfacial trauma), it may not fully reflect the extent or complexity of the injury. Even though the ODI scale was designed to be objective, the evaluation of fractures according to ODI scores may still involve some subjectivity, particularly in borderline cases or in the presence of unclear imaging findings. Importantly, interrater reliability was not assessed, and the consistency of scoring between different observers remains unknown. Future research should evaluate the scale’s reproducibility among clinicians with varying levels of expertise to ensure its objectivity and clinical applicability.

### 4.7. The Clinical Application of the ODI Classification System

#### 4.7.1. Selection of Treatment Method Based on ODI Classification Results

As previously mentioned, surgical treatment based on ODI scale values corresponds with treatment decisions derived from anatomical classifications. Thanks to the standardized, simple, and numerical nature of the ODI classification, maxillofacial surgeons can quickly and accurately assess both the location and severity of the orbital trauma, which facilitates the prompt selection of an appropriate treatment method especially in severe cases of maxillofacial trauma.

#### 4.7.2. Broad Implementation of the ODI Classification Across Medical Disciplines

This ODI classification system stands out as an easy-to-use tool that could be successfully administered across different specialties. Wider adaptation of the ODI classification could improve communication between specialists from various medical fields, and its universality might allow it to be used worldwide. This system is likely to be used by emergency physicians, traumatologists, radiologists, plastic surgeons, and forensic medicine doctors, who may not have advanced training in maxillofacial surgery and lack proper expertise. This multidisciplinary approach is especially valuable in emergency settings, where timely and accurate decisions are critical.

In addition, the ODI serves as a valuable educational tool for junior maxillofacial surgery residents. For those still developing clinical judgment and anatomical knowledge, the clarity and structure of the ODI system can help bridge the gap between theoretical knowledge and practical application. By guiding less experienced clinicians in recognizing key fracture patterns, the scale reduces the likelihood of diagnostic or treatment errors and supports more accurate selection of treatment strategies.

#### 4.7.3. Improvement in Patient Care

The ODI classification could also promote a more standardized approach towards documenting and comparing cases across different institutions and countries. This could, in turn, contribute to more unified global standards in managing orbital trauma. Adopting this system might improve interdisciplinary communication and reduce diagnostic delays, ultimately leading to better treatment planning and patient care. By enabling early assessment of orbital trauma and selection of the most suitable surgical interventions, the ODI classification might help reduce unnecessary procedures, shorten hospital stays, and ultimately contribute to more cost-effective treatment of orbital injuries without incurring unnecessary hospital costs.

#### 4.7.4. Combination of ODI and Artificial Intelligence (AI)—The Future of Diagnosis of Orbital Fractures?

Artificial intelligence is currently one of the leading topics in diagnostic imaging, including orbital fracture imaging. It is increasingly emphasized that the assessment made by a radiologist remains a subjective method with a certain risk of error [38]. Artificial intelligence (AI) has the potential to revolutionize diagnostic imaging in radiology by supporting physicians, improving the quality of described examinations, and streamlining reporting and workflow [39,40].

The ODI classification holds great potential for the development of AI-based tools for detection, classification, and treatment decision making of orbital fractures. Due to its numerical and standardized nature, the ODI score provides an ideal framework for training machine learning models. Those programs could be developed to automatically identify fracture lines, segment the orbital anatomy, and calculate displacement values, ultimately generating an ODI score without the need for manual data entry. This shows great promise to increase the speed and accuracy of diagnosis, especially in the setting of hospital emergency departments.

### 4.8. Future Perspectives

As one of the few research groups working on this topic, we decided to investigate the usefulness of this new, yet promising scale by enriching the scientific community with the latest data. Although the ODI classification may be helpful for quicker diagnosis and management of orbital fractures, its adaptation is not yet widespread. Further studies involving larger patient groups, including a broader range of orbital trauma cases, conducted across multiple countries, are needed to confirm its reliability in diverse clinical settings. Additionally, evaluating the use of the ODI scale among maxillofacial surgeons and other medical specialists could determine its broader adaptation and potential benefits across different fields of medicine. Wider adoption may also contribute to standardizing trauma assessment and improving interdisciplinary decision making.

## 5. Conclusions

Orbital injuries can be a clinical challenge for primary care physicians and emergency department doctors—non-head-and-skull specialties—in terms of their diagnosis, treatment plan, and prognosis. Understanding their etiology, symptoms, and complications is essential to implement appropriate treatment strategies to have high and immediate pro-health effectiveness. The ODI classification can be a useful topographic/morphological–therapeutic classification when making a diagnosis during admission, which can certainly replace the use of typically anatomical classifications. Assigning a patient to a given value indicates severity of trauma, the probable type of treatment needed, and may indicate probable accompanying symptoms. Further multicenter studies with larger numbers of participants are necessary.

## Figures and Tables

**Table 1:** Conversion of the diagnosis based on Wanyura’s classification to the anatomical classification.

Initial Classification	Converted Classification
ZJSO	Zygomaticomaxillary complex fracture (ZMCO)
ZIDO	Isolated orbital floor fracture
ZCO/ZCON	Fronto-orbital fracture
Le Fort	Le Fort (no change)

**Table 2:** ODI scale.

ODI Scale	Description	Abbreviation
1	Destruction of the floor, involving one wall	1W
2	Destruction of the floor plus one wall (medial or lateral)	2W
3	Destruction of the floor plus one wall and one orbital margin	1W + 1M
4	Destruction of the floor plus two walls and one orbital margin	2W + 1M
5	Destruction of the floor plus one wall and two orbital margins	2W + 2M
6	Destruction of the floor plus two walls and one orbital margin	3W + 1M
7	Destruction of the floor plus one or two walls and two orbital margins	3W + 2M
8	Destruction of the floor plus two or three walls and more than one orbital margin	3–4W + 2–4M

W—walls, M—margin.

**Table 3:** Eligibility criteria.

Inclusion Criteria	Exclusion Criteria
Complete patient history available at the Maxillofacial Surgery Department	Incomplete medical records
ICD-10 codes—S02.3 and S02.4	No orbital fracture
Available radiological examination or its full description	
Patients treated primarily at the Maxillofacial Surgery Department	

**Table 4:** General information for included 160 cases.

Variable	Age	ODI Diagnosis	Timing to Register	Hospitalization Duration
Average	40.0 ± 17.5	2.9 ± 1.7	0.9 ± 2.5	3.3 ± 1.2
Median	37.5	3.0	0	3.0
Minimum	13.0	1.0	0	1.0
Maximum	90.0	8.0	19.0	8.0
Range	77.0	7.0	19.0	7.0

**Table 5:** Distribution of the type of treatment undertaken.

Type of Treatment	Number of Patients	Percent (%)
Surgical	138	86.3
Conservative	22	13.8

**Table 6:** Distribution Of Methods Of Treatment Among Surgically Treated Patients.

Type of Treatment	Number of Patients	Percent (%) of Surgically Treated Patients
Plate Osteosynthesis	62	44.9
Wall Reconstruction	45	32.6
Closed Reduction	25	18.1
Plate Osteosyn. and Wall Recon.	6	4.3

## Data Availability

The data presented in this study are available on request from the corresponding author due to patients’ privacy.

## Abbreviations

The following abbreviations are used in this manuscript:

ODI	Orbital Destruction Intensity
ZMCO	Zygomaticomaxillary complex
NOE	Naso-orbito-ethmoid
ZCON	Złamanie czaszkowooczodołowonosowe (cranio-orbito-nasal fracture)
ZIDO	Złamanie izolowane dna oczodołu (isolated orbital floor fracture)
ZJSO	Złamanie jarzmowoszczękowooczodołowe (zygomaticomaxillo-orbital fracture)
ZJO	Złamanie jarzmowooczodołowe (zygomatico-orbital fracture)
CT	Computed tomography
MRI	Magnetic resonance imaging
WL	Window level
WW	Window width
W	Walls
M	Margins
